# Power and Integrated Health Care: Shifting from Governance to Governmentality

**DOI:** 10.5334/ijic.2480

**Published:** 2016-09-14

**Authors:** André Janse van Rensburg, Asta Rau, Pieter Fourie, Piet Bracke

**Affiliations:** 1 Department of Political Science, Stellenbosch University, ZA; 2 Department of Sociology, Ghent University, BE; 3 Centre for Health Systems Research & Development, University of the Free State, ZA

**Keywords:** Governance, power, integrated care, governmentality

## Abstract

Integrated care occurs within micro, meso and macro levels of governance structures, which are shaped by complex power dynamics. Yet theoretically-led notions of power, and scrutiny of its meanings and its functioning, are neglected in the literature on integrated care. We explore an alternative approach. Following a discussion on governance, two streams of theorising power are presented: mainstream and second-stream. Mainstream concepts are based on the notion of power-as-capacity, of one agent having the capacity to influence another—so the overall idea is ‘power *over*?’. Studies on integrated care typically employ mainstream ideas, which yield rather limited analyses. Second-stream concepts focus on strategies and relations of power—how it is channelled, negotiated and (re)produced. These notions align well with the contemporary shift away from the idea that power is centralised, towards more fluid ideas of power as dispersed and (re)negotiated throughout a range of societal structures, networks and actors. Accompanying this shift, the notion of governance is slowly being eclipsed by that of governmentality. We propose governmentality as a valuable perspective for analysing and understanding power in integrated care. Our contribution aims to address the need for more finely tuned theoretical frameworks that can be used to guide empirical work.

## Introduction

The study of integrated care, how it is governed and the complex interrelations that constitute it have been the subject of numerous scientific investigations and policy reforms. Integrated care unfolds within distinct governance structures [1], across micro, meso and macro levels of analysis [2,3]. Pressures to democratise decision-making processes have led governments globally to place increasing emphasis on partnerships in health care delivery [4]. Health care-related activity is very much subject to politics [5], and a clear and comprehensive understanding of power is necessary in order to “build-up rich and nuanced descriptions of the forms, practices and effects of power” in integrated care and its governance [6, p. 367]. But studies on integrated care that focus on power rarely base their discussions on properly theorised notions of what power is and how it functions. To begin exploring how this gap can be addressed, a brief outline of two broad trends in theorising power is presented. This article proposes that conceptual applications of governance to integrated care are limited in that they under-emphasise types of governance built on more fluid and subtle (as opposed to more determinist and direct) understandings of power. The article then introduces and explores as an alternative to governance, the notion of governmentality, which incorporates more subtle and contemporary understandings of power. We argue that the shift in emphasis from governance to governmentality could address the need for more finely tuned theoretical frameworks address power and to guide empirical work on integrated care.

Before proceeding to the sections on governance and power, let us briefly clarify what is meant by *integrated care*. *Integrated health care* (or integrated care) in health systems is a collection of strategies encompassing patient-centred, demand-driven, multi-level, and multi-modal (multiple methods/ways of) collaborative processes among various professionals, organisations and sectors towards coordinated patient care [7,8,9]. Integrated care has become a well-established feature of national, regional and global health policy. It is an increasingly popular strategy to address fragmented and uncoordinated health systems [10], as well as to increase accessibility to care (especially of disadvantaged communities) [8,11]. Its focus on continuity of care, service partnerships and patient-centeredness has been attractive to health reformers, and it is widely-recognised for its attention to patient needs. Research on the topic has grown exponentially, and its development as a public health concept and strategy is underpinned by concerted efforts towards better understanding the complexities and difficulties associated with integrated care [12,13,14,15]. This journal has especially been at the forefront of outlining the meaning and scope of integrated care. Most notably, these efforts culminated in the development of the Rainbow Model of Integrated Care by Valentijn and colleagues [2,3]. The Rainbow Model provides a fitting snapshot of the complexity and range of integrated care, describing its forms across micro, meso and macro domains.

## The governance of integrated care

*Health governance* essentially refers to rules that govern the roles, responsibilities and interactions among service users, government decision-makers and the health service providers. These interactions ultimately shape the social organisation of health care, namely public, private and non-profit [16,17,18,19]. *In defining health governance*, consideration must be given to the shift from ‘government’ to ‘governance’. This shift denotes a modification from public administration as a homogeneous central state that provides services to a passive public via expert professionals, towards one where the state is but one part of a “mixed economy of funding and provision”, which also includes active public consumers and increased managerial control over expert professionals [20, p. 159]. Health governance does not necessarily refer to management, but rather to continuous processes of strategic interaction and negotiation among health care stakeholders at various levels [21].

The success of integrated care is significantly tied to the degree of stakeholder collaboration and the extent to which different care components are governed [22,23]. Governing integrated care can be distilled into three levels. On the micro level, inter-professional or clinical governance takes place, on the meso level inter-organisational governance occurs, while on the macro level the ideal of *good* governance is shaped and pursued by the collective efforts of large multinationals—for instance in the design and application of global indicators to shape and monitor progress towards good governance values [3].

### Micro level governance

Inter-professional governance focuses on “openness, integrity and accountability between professionals at the operational level (e.g. joint accountability, appeal on pursued policies and responsibilities)” [3, p. 8]. Inter-professional collaboration is hampered by a range of factors, including poor communication, conflicting power relations and role confusion [24]. Against a backdrop of increasing variability in terms of leadership, culture, participation and professional status, both between and within specialities of professionals that have to align to cater to individual patient needs [25], the burden of collaboration and coordination in clinical settings has been shifted to health professionals [23]. This gave rise to models of shared governance, a key part of collaborative practice among professionals [26], and a useful mechanism with which to redistribute traditional clinical authority, responsibility and accountability [27]. Many such models exist, and have especially been growing in popularity in North America [26,27,28,29]. An example is the establishment of Inter-professional Practice Councils (IPPCs), a model underpinned by collaborative, multidisciplinary decision-making and shared accountability for care quality and safety by frontline workers [29].

A related concept is clinical governance, introduced in the United Kingdom’s National Health Service during the late 1990s. It surfaced within a broader contexts of the rise of clear financial accountability, the amplification of cost-effectiveness in health care, the crystallisation of service provision needs assessments, and the emergence of the “evidence-based medicine” paradigm [30]. Clinical governance was designed to overcome traditional power struggles in multidisciplinary team-working [31], and to “consolidate, codify, and universalise often fragmented and far from clear policies and approaches”, shifting final accountability and responsibility for clinical practice to senior clinicians [32, p. 62]. It explicitly recognises the centrality of clinicians to the performance and organisation of clinical work and provides clinicians with a medium for integrating the clinical, resource, and organisational aspects of care [33]. Clinical governance has been used in the professional integration of mental health care by fostering collaboration between multidisciplinary teams and primary care health professionals by having shared referral, assessment, and management guidelines [34].

### Meso level governance

Certainly one of the most widely studied forms of integrated care is organisational integration, and it is here that the conceptualisation of governance has been most prominent [2]. Principally, three modes of governance have been defined: *hierarchy* (command is the basic mechanism of control and coordination); *market* (price-driven transactions between consumers and providers as the key coordination mechanism); and *network* (coordination by means of mutual, trust-driven contact, negotiation and adjustment). These three modes differ in terms of the positioning and influence of the stakeholders involved, and therefore in terms of the distribution and dynamics of power. In reality, these ideal modes rarely (if ever) occur in isolation; rather, hybrid forms of governance emerge, and this presents an additional level of complexity to understanding the dynamics of power [4,35,36]. Additionally, collaborative partnerships progress through life cycles, each of which may be characterised by one or more different forms of governance, which implies different power dynamics over time [4]. Little research has focused on different modes or forms of meso-level governance, power and integrated care. One example suggested that—in terms of integrated care development—England tended to exemplify more hierarchical modes of governance, in contrast to the Netherlands’ more network-based forms, each with its own consequences for the different relations and manifestations of power [22].

Network governance is being paid increasing attention due to the collaborative nature of integrated care. Network governance is defined as the coordination of the collective action of contracted public and private organisations that provide public services [37]. Ahead of our later explorations on the meaning and functioning of power, it needs to be noted here that the idea of collectives and collective action implies social power—in other words power vested in or enacted by groups, or by individuals as group members. Different types of network governance have been identified. These network governance types have been differentiated in terms of coordination and exchange, such as mutual adjustment, alliance and corporate structure [38]; in terms of differences in centrality and density, for instance participant, lead organisation and network administrative organisation forms [39]; and in terms of the partners involved and their relative levels of participation, for instance government-led, clustered participatory and hybrid public-private collaborative forms of network governance [40]. Research studies employing theorised network governance types include Wiktorowicz et al. [37], who distinguished different forms of social power among different mental health care networks in terms of rural/urban and regionalised/non-regionalised dichotomies, and Fleury et al. [41], who underlined the consequences of social power as exercised in corporate and alliance governance forms within integrated mental health networks.

### Macro-level governance

Systems-level or macro-level governance involves the creation of “trust towards external stakeholders (e.g. municipality and health insurance companies) based on working method, reputation, management, control and/or supervision” [3, p. 8]. Ultimately, in order to achieve an integrated system of care, “governance needs to be aligned across the various health and social care providers to drive shared interests and accountability in care delivery for people across hospitals, community services, general practice teams and social care” [42, p. 9]. The body of work on ‘good governance’ on a systems level has been driven forward by international, regional, and national reports on progress towards good governance—based on globally agreed (if not negotiated) indicators, data collected against those indicators, and analyses.

Influential global bodies have been instrumental in forwarding more normative conceptualisations of ‘good governance’ (i.e. normative in terms of Western values and aspirations). The World Health Organization’s guidelines for better stewardship [43] and toolkit for the monitoring of health systems governance [44]; World Bank governance guidelines and indicators [45,46]; and the United Nations Development Programme’s principles of good governance [47] are examples of influential bases from which subsequent frameworks were developed. Particularly health system governance frameworks geared towards low-to-middle income settings [48,49,50,51] have received focus.

Also building on World Bank conceptions, Brinkerhoff and Bossert [18,19] put forward a health governance model that features key categories of health system actors (state, providers, and clients/citizens); their model differs somewhat from predominantly system-focused frameworks by employing a distinctly relational epistemology that stresses the centrality of the connections among the three groups of actors. This approach is very much tied to the central nodal relationships in health system governance, namely state and market; health ministries and other ministries; public sector, civil society and the private sector; the health system reform spectrum from static to dynamic; and health reform and human rights-based approaches to health [48]. These normative frameworks, although relatively recent, have proven useful to analyse the role of district health governance on integrated primary mental health care [52].

The conceptualisations of governance outlined thus far provide us with insight into the ways in which integrated care is strategized and structured. Power is central to the ways in which governance is structured and operates [18,36,53,54,55]. Both governance and integrated care essentially entail relations among diverse actors, with different capacities, agendas and interests. Whether the governance of integrated care occurs at the professional [56,57,58], organisational [22,36,58] or system level [52,59], power is a central concern. This said, power is poorly defined in studies on governance and integrated care, and the term is often used ambiguously and without due consideration of the potential complexities it contains. The next section will therefore pay attention to theoretical understandings of power, as well as to the ways in which the concept is applied in studies on integrated care.

## A brief outline of past and current trends in understanding power

Scott [60, p. 25] offers a solid starting point for an exploration of power by pointing out that “power can be understood, at its most basic, as being the production of causal effects.” Most power theorists would readily accept this claim, but beyond that their views diverge into many different streams of thought as demonstrated in the extensive accumulated body of knowledge driven by a multitude of disciplines and scholars. As Wrong [61, p. viii] aptly notes, power is “an essentially contested” concept. An overview of the many different developments and theories of power is not the aim of this article, and at any rate, is offered elsewhere [61,62,63,64]. Rather, we set out to map some *main* currents of thought in order to identify those that may best apply to studying governance of integrated care. The literature points to two streams of thought and research on power, namely, *mainstream* and *second stream* interpretations [62,63].

### Mainstream understandings of power

Mainstream thought focuses on sources of power and is rooted in the idea of power being exercised by one agent over another [62]. This flows from early ideas developed by Thomas Hobbes, and which focused on *what power essentially is* [63], whether “Originall or Instrumentall” (i.e. natural or instrumental powers of individuals), Social (i.e. collective power), or Sovereign (i.e. created by the transfer of individual rights to one or several people, with the idea that individuals will have their general protection guaranteed) [65, p. 66]. A major proponent of mainstream tradition was Max Weber, who viewed the state and its related bureaucracies as key sources of power, and defined power (*Macht*) as “the probability that one actor within a social relationship will be in a position to carry out his own will despite resistance, regardless of the basis on which this probability rests” [66, p. 139]. Weber’s conception of power was furthered by Robert Dahl [67,68] whose ideas became a common starting point for the study of power during the second half of the 20th century [69] and remains a popular basis from which contemporary scholars launch newer ideas. For instance, Dennis Wrong adds to mainstream understandings by including a focus on ‘power *over*’ to allow for more subtle and hidden facets of power. Wrong [61, p. 2] defined power as “the capacity of some persons to produce intended and foreseen effects on others”. He stressed that the conceptualisation of power needs to be posited as something intentional, effective, and include a distinction between latent and manifest forms of power.

One of the first major theorists whose work signalled a shift from Weber’s ideas was Steven Lukes [64]. He argued that institutional practices and social forces do not enter politics necessarily through individual action. He proposed three ‘faces’ of power: decision-making power (political action), non-decision-making power (covert and overt agenda setting), and ideological power (which offsets the predominantly behavioural focus of the first two, and allows for an analysis of latent and observable conflicts in worldviews). Lukes’ work is clearly embedded in mainstream concepts, but in later writings we begin to see a growing attention to the role of social structures in power and the exercising of it.

### Second-stream understandings of power

Second stream power scholars share some aspects of mainstream thought, but break significantly with the idea of ‘power *over*’ and mainstream’s emphasis on *sources* of power—to focus on processes, techniques and strategies of power [62]. Deleuze [70, p. 70–71] argues that we should not ask “What is power and where does it come from?’, but ‘How is it practised?”, noting that power means “to incite, to induce, to seduce, to make easy or difficult, to enlarge or limit, to make more or less probable”. Built on Machiavellian [71] notions on *what power does* [63], the second-stream tradition centres on how power is established and re-produced within a network of relations in political-strategic ways [72].

Most second-stream scholarship on power originates in, or is a response to, the revolutionary ideas of Michel Foucault—the most influential theorist on power in the late 20^th^ Century. His writings link relations of power to the construction of knowledge and identity. In turn he links these notions to processes of governance and discipline—both of society and of the self [72,73]. Foucault’s work demonstrated how norms and structures become established and entrenched (institutionalised) throughout history via relations of power, and how these norms and structures shape (construct) the identities of individual and social actors. He also showed how—in a series of slow cyclical processes—the actions of individual and social actors then feed back into those very same norms and structures and in doing so *re-*shape them, sometimes in surprising ways. In these cycles of construction and re-construction, people and groups (subjects) become positioned in specific ways in relation to each other and in relation to dominant norms and structures. Foucault views relations of power as extending well beyond the limits of the state, the state being superstructural to a range of different networks of power that weave throughout society [74].

This interest in the mutually constitutive relationships of power between (a) structures, (b) the norms whereby structures become entrenched and institutionalised, and (c) individual/social actors—is also reflected in the work of sociological giants like Pierre Bourdieu and Anthony Giddens. Compared to Foucault’s keen focus on processes and relations of power, however, they place much more emphasis on the structures within which power is enacted. Bourdieu’s work centres on four key *sources* of power—economic, cultural, social and symbolic capital. But his is also a ‘theory of practice’ in that he explores how these sources of power are mobilised and operate via *habitus*—a set of dispositions and meanings that people gain through socialisation—within structured social fields [75,76,77]. Giddens, in his structuration theory [62] also concentrates on the tension between structure and agency. He sees power as comprising “reproduced relations of autonomy and dependence in social interaction” [78, p. 39]. And he emphasises that social interaction cannot be analysed apart from the social structures within which they take place. In his view people are free to act, but draw upon and tend to replicate structures of power through their own actions.

John Scott [62,60], noting the divergence between mainstream and second stream thinking on power, attempted to systematically bring together elements from both. Scott’s conception distinguishes two groups of “elementary forms of power” [62, p. 1]: (1) *corrective influence* includes force (negative, physical sanctions that prevent subalterns’ actions—subalterns being of subordinate or inferior position) and manipulation (various kinds of both positive and negative sanctions that influence subalterns’ intentions), and (2) *persuasive influence* includes signification (persuasion by means of cognitive symbols such as text-based ideas and representations) and legitimisation (persuasion through building value commitments to certain ideas and ideals). Remarking that such a synthesis is a “fundamental priority”, Scott [62, p. 12] argues that his theory unifies the two streams of power.

Steward Clegg drew from both mainstream and second stream ideas on power to construct his “circuits of power”, which represents the different ways in which power flows at different levels. Using a metaphor of power moving through an electric circuit board, three multi-level, distinct and interactive circuits through which power must necessarily flow: episodic (micro level), dispositional (macro level), and facilitative (macro level). The episodic circuit represents micro-level and irregular exercises of power by agents in response to everyday interactions. On a macro-level, the dispositional circuit represents socially constructed meanings and rules, while the facilitative circuit signifies technologies, networks and environmental factors that punish or reward episodic circuit agency [63,79].

An important goal of second-stream power theories is to uncover, and by implication help address, structures and processes that disenfranchise some people or groups in favour of empowering others. Essential to this ethic is Antonio Gramsci’s concept of hegemony, which occurs when dominance becomes entrenched via the reproduction of norms favoured and promoted by dominant groups, called elites. In this process dominant classes gain and keep the consent of the (subaltern) majority without relying on any direct forms of compulsion or subjugation [62]. In this view “force will appear to be based on the consent of the majority, expressed by the so-called organs of public opinion newspapers and associations which, therefore, in certain situations, are artificially multiplied” [80, p. 80]. An ominous aspect of this artificiality is that people become complicit in the value systems of dominant groups to the extent that they act—knowingly or unknowingly—in the interest of the powerful. Hannah Arendt’s contributions to scholarship on power are particularly influential in opposing hegemony. She distinguishes power from violence, strength and force, and views power as the product of collective action of actors bound together in a common political purpose, and based on rational persuasion and consent rather than coercion [81].

## The study of power in the literature on integrated care

The importance of understanding the different forms of integrated care governance and its associated dimensions of power has not been ignored by researchers. In fact, several studies have focused on the interplay between governance, integrated care and power. For instance, Fleury et al. [41] examined the effectiveness of a managerial tool in changing health care, more specifically, the impact of regional planning implementation processes on the creation of integrated mental health service networks. The findings suggested that alliances between organisations are negotiated forms of power, and that certain governance types foster decision-making and influencing powers for certain actors. In studying the influences of the public-private mix in social care systems in the Netherlands and England in the development of integrated care, Mur-Veeman et al. [22] suggested that centralised, hierarchical governance illustrate different forms of power than more networked, dispersed governance systems. Rodríguez et al. [36] examined the values, interests, and mobilisation of power within available governance networks of organisational actors in three collaborative initiatives. The authors found power dependencies in inter-organisational relationships in terms of formal authority, control of critical resources, and discursive legitimacy, reproduced over time. Wiktorowicz et al. [37] explored the governance processes and supporting conditions that foster inter-organisational collaboration in mental health networks. The study theorised forms of power associated with different mental health network governance models, namely, authority, negotiation, and influence.

Studies on integrated care and its associated forms of power have yielded different understandings of power: power as capacity [82]; power as resource [83]; power as strategy [84]; and what Scott [62, p. 16] refers to as “structures of domination” [22]. Some studies [36,85] rely on conceptions of power as wielded by certain actors, who reside in certain positions or have a certain status, which they use in order to further their interests. This predominantly mainstream approach is particularly found in research on collaboration among integration-related role-players, such as medical and non-medical actors. For example, Tousijn [83, p. 523] describes the identification of power relations in multi-professional teams as a major barrier to integration, referring to studies that have especially focused on “the dominant position held by the medical profession; the propensity of each profession to defend its own jurisdiction; and the existence of different professional cultures and values, which generate inter-professional tensions”.

Similar tensions have been explored in network research. Essentially, actors’ positions within a network places certain constraints on and provides certain opportunities for their potential to bargain and negotiate, thereby creating different bases of power [86,87]. In network thinking power is inherently relational, in the mainstream sense of ‘power *over*’: a person or organisation has power because they can dominate others (who in turn are dependent on them). However, power can also be systemic, in that power is more easily exercised in more dense networks. Therefore power in networks can both refer to the relations among individual actors, and to a description of a population. One of the most common social network measures is network centrality. Centrality aims to identify actors who are in a position of privilege (and therefore power) relative to actors in more peripheral positions in a network [88]. Though not always explicitly presented as a measure of power, centrality has been used in studies on integrated care, especially in professional or inter-organisational collaboration [89,90,91,92,93,94].

In short, studies on power in integrated care have leaned towards its mainstream conceptions. A search of the existing literature yields little research on integrated care conducted with the more subtle, relational, second stream understandings of power. Gilbert [56] drew from a Foucauldian biopolitics perspective in order to explore the relationships between policy, professional practice and the people who are the subjects of that practice. Using Foucault’s notion of governmentality, Ferlie et al. [95] analysed network organising in the UK cancer field. The idea of governmentality and the ways in which it unfolds in integrated care holds much promise, and is yet to be fully examined. As we hope the discussion on key theories of power has demonstrated, power is a multi-layered, complex construct, which involves far more than relations among key role-players (although this remains a salient part of integration processes) [62,63]. Adding to this complexity is the different levels and modes of governance that steer integrated health care. Mainstream perspectives on power in integrated health care [22,36,82,85] unfortunately only provide a limited view. We now present a discussion proposing existing models from governmentality studies hold the potential to resolve the lack of second stream power understandings in both integrated health care and its governance.

## From governance to governmentality of integrated mental health care

The idea of governmentality is inspired by Foucault’s later writings—and firmly embedded in second-stream notions of power. Underlying the idea of governmentality are the ways in which people are influenced to govern themselves, a notion of power that is dispersed throughout a population. Governmentality allows for governing at a distance, by embodying discipline in individuals through the creation of docile agents to be used in modern political and economic institutions. Essentially, when people/groups embody the norms in which they are embedded, they *self-regulate* their actions, their perceptions and even their values according to those norms—in other words, they self-regulate.

In step with this, governmentality perspectives identify and analyse “the complex of rules, norms, standards, and regulatory practices that extend state rule more deeply into civil society by regulating the ways in which civil society self-regulates” [54, p. 62]. The focus shifts away from state-centred governance, towards self-regulation and reflexivity that are rooted in governance regimes that influence individuals to behave in a certain way [17]. A governmentality perspective starts from the standpoint that governance is made up of inter-dependent organisations that together form “semiautonomous and self-governing networks” [54, p. 62], denoting shifts from traditional, Weberian, bureaucratic forms of governance, to more indirect, network-type forms, relations and processes [96,97]. The notion of governmentality does not necessarily privilege the state as locus or origin of power, but takes self-governing practices as starting point—this allows a mapping of multiple centres of calculation and authority that traverse and link up personal, social and economic life [98]. The focus falls on “power without a centre, or rather with multiple centres” [98, p. 9]. Individual freedom is not an opposing feature of power, but rather a salient part of its operations; power is not about constraining individuals, but rather about creating people who are “capable of bearing a kind of regulated freedom” [99, p. 174].

Given its conception of power as relationally dispersed rather than focused in the state, governmentality in integrated care highlights the importance of non-state actors such as non-governmental organisations and private practitioners. We concur that the dichotomies through which power has been traditionally characterised—such as state versus civil society, public versus private, public versus private, and coercion and consent—and the mainstream concepts of power that underlie these dichotomies—do not provide an adequate understanding of the ways in which power operates [99]. Rather, the focus should fall, as it does in the notion of governmentality, on the “technologies of the self”—the ways in which individuals, or groups, shape the behaviour of others and of themselves. And this includes the “complex of practical processes, instruments, programs, calculations, measures, and apparatuses making it possible to form and control forms of action, structures of preference, and premises for decisions by societal agents in view of certain goals” [100, p. 12].

While a governmentality perspective certainly opens up interesting and useful avenues of exploration, it has not been exempted from critique. It has been argued that a governmentality approach ignores lay normativities in everyday routine interactions and is unable to take into account the practical resources through which power operates [101]. Others have noted that a governmentality approach foregoes its critical and emancipatory potential in exchange for a theory of social reproduction, in that the approach focuses on a conception of power that externalises and marginalises contradiction and struggle [102]. These are valid concerns, and those who pursue the study of integrated care through a governmentality angle should engage with such criticisms. Nonetheless, governmentality as a theoretical construct offers much to integrated care scholarship, as many examples in existing literature highlight.

A governmentality perspective has found great appeal in fostering better understanding the nuances of clinical governance [20,56,57]. The approach is flexible enough to allow space for other perspectives, for instance, combining governmentality studies with Courpasson’s “soft bureaucracy” in the study of clinical governance [20]. A governmentality approach can open up the more subtle ways in which power work in different settings. It has been used to show how the technology of psychology has been employed as a strategy of government in post-apartheid South Africa [103]. It has been shown to be useful in exploring how multidisciplinary mental health outreach teams are managed “at a distance” through subtle “deep management” practices [104]. Further, a governmentality perspective has been used to theorise the ways in which psychiatric nurses govern correctional inmates with mental illness, specifically, by means of sovereign, disciplinary and pastoral power [105]. Sending and Neumann [106, p. 668] critiqued global governance processes as it is presented in existing literature, namely that state and non-state relations is a zero-sum game concerned with the “triad between sovereignty, authority, and legitimacy”. The authors used a governmentality lens to study the “rationalities of government” and showed that civil society is often made up of political subjects whose autonomy and expertise are crucial elements of governing, that governing occurs through autonomous subjects and not passive objects.

Given its increasing popularity and promise, what can a governmentality approach offer to the study of governance and power in integrated care? Several key areas of investigation emerge, and given the wide array of health system configurations and contextual factors surrounding integrated care, the research possibilities are truly wide-ranging. This said, two areas of interest can especially be fruitful in unpacking the governance of integrated care. Firstly, a governmentality perspective can accentuate the “technologies of the self”, the ways in which the behaviour of those involved in integration processes are normalised, disciplined, empowered and sanctioned. Understanding how the energies of those involved in integrated care are governed – be it clinicians, governors or patients – can potentially emphasise how power operates in different settings. Such an approach also does not position integrated care as a politically neutral project, but as one fraught with processes of both overt and subtle domination. The second suggestion relates to the make-up of health system configurations. Depending on the country context, integrated care unfolds to varying degrees in accordance to the relations between state and non-state entities. For instance, in West European countries such as Belgium and the Netherlands the state has a more facilitating role, leaving the provision of health services to various non-state service organisations. In Southern African countries however, health services are mainly the purview of the state, and is augmented by different non-state organisations. In most contexts however, the state is the main steward of health care, suggesting a dominating, sovereign role in integrated care. However, a governmentality perspective permits us to move beyond traditional governance dichotomies such as state versus civil society, public versus private, public versus private, and coercion and consent [99]. A governmentality view allows us to understand the ways in which governance relations between state and non-state service providers play out in integrated care configurations, providing insight into the more subtle ways of governing and emergence of power. This is a potentially rich area of investigation, especially in the contexts of wide-spread neoliberal health care reform where power has been reduced to much more indistinct strategic processes [99].

At this point it is important to note that the argument forwarded in this article has not been that the study of conventional forms of governance and power in integrated care should be substituted by a Foucauldian governmentality perspective. Rather, our key argument is that the more subtle, second stream of power research has been neglected in integrated care governance research, and that a governmentality perspective can open up helpful avenues of investigation in this sense. In describing the incomplete and fractured nature of our knowledge of integrated care, Kodner [8, p. 12] notes that “in some ways, we are like blind men and the proverbial elephant, each aware only of the part of the animal touched and with no experience of the whole”. In line with this metaphor, we stress Scott’s [62] sentiment that different understandings of power should not be viewed as opposing perspectives, but rather as complementary. In a similar way, governmentality studies should not be seen in opposition to more normative understandings of governance. Rather, it should be seen as complementary, providing us with a diagnostic insight rather than the descriptive leanings of “the sociology of governance” [107, p. 16]. Ultimately we should drive integrated care scholarship forward in a comprehensive, inclusive way, reflexive and open to critique.

## Conclusion

The existing and ever-expanding literature on the concepts under scrutiny in this article—integrated care, governance, and power—is diverse and voluminous. From this oeuvre we extracted, and outlined, different modes in the governance of integrated care, alongside two main streams/traditions of power. We then summarised some of the ways in which governance and power have been applied in studying integrated care. We argued for the value of second stream concepts of power over the limitations of mainstream concepts of power. We then show how the notion of governmentality links to second-stream thinking on power and propose governmentality as a useful perspective from which to advance and enhance current understandings of governance and power in integrated care.

The popular appeal of integrated care in health reform agendas has increased scrutiny on its governance, and rightly so. Governance underwrites the outcomes of integrated care in a fundamental way, and provides a gateway through which we can better understand the processes and politics that influence these complex dynamics. Grasping the ways in which power is present in the relations that constitute integrated care and its governance is key to comprehend the reasons why integrated care is often such a challenging ideal to achieve. To this end we argue that there is a need to go beyond traditional governance models and their inherent conceptions of power, towards critically examining the subtle and subvert ways in which integrated care is steered. This, we propose, can be achieved by focusing on the study of governmentality. The ultimate aim of the article is to add to the construction of a more comprehensive, nuanced and rounded understanding of integrated care and its mechanisms.
